# Association between fasting serum uric acid levels and Alzheimer's disease in nursing home residents: a cross-sectional study

**DOI:** 10.3389/fnut.2026.1878852

**Published:** 2026-08-06

**Authors:** Xiaoyu Lin, Wenjian Zhao, Jinchi Zhou, Jiaqi Qiu

**Affiliations:** 1 Department of Neurology, Guangzhou Geriatric Hospital, Guangzhou, Guangdong, China; 2 Center for Child and Adolescent Psychological and Behavioral Medicine, Shandong Mental Health Center, Shandong University, Jinan, Shandong, China; 3 Department of Internal Medicine, Guangzhou Geriatric Hospital, Guangzhou, Guangdong, China

**Keywords:** Alzheimer's disease, cognitive impairment, cross-sectional studies, fasting serum uric acid, older adults

## Abstract

**Introduction:**

Evidence regarding the association between fasting serum uric acid (FSUA) and Alzheimer's disease (AD) is limited and inconsistent. This study investigated the association between FSUA levels and AD.

**Methods:**

In this cross-sectional study, 852 participants from a hospital specializing in geriatric care were enrolled. Multivariable logistic regression analysis, restricted cubic spline curves, and subgroup analyses were employed to analyze the association between FSUA and AD.

**Results:**

The average age of eligible participants was 81.0 ± 8.8 years; 23% were patients with AD and 33.7% were male. Multivariate logistic regression analysis revealed an inverse relationship between FSUA levels and AD. Specifically, a 10 μmol/L increase in FSUA was associated with a 5% decrease in AD odds (OR, 0.95; 95% confidence interval [CI], 0.93–0.97). When FSUA levels were divided into quartiles, the higher quartiles showed progressively lower odds of developing AD than those in the lowest quartiles (*p* for trend < 0.001). No interactions were observed between FSUA and AD. Restricted cubic spline regression model demonstrated a linear inverse association between the odds of AD and FSUA levels.

**Conclusion:**

We observed a negative association between FSUA levels and AD. Further prospective studies are required to confirm this causal relationship.

## 1 Introduction

Alzheimer's disease (AD) is the leading cause of dementia and is a major and growing public health issue worldwide, especially among fast-aging populations in nursing homes (1–3). These individuals frequently exhibit complex comorbidities and receive polypharmacy, which may influence the odds and progression of AD compared with their community-dwelling counterparts. Consequently, the identification of modifiable risk factors or biomarkers within this population is vital for the formulation of specific preventive measures and the improvement of care standards. Fasting serum uric acid (FSUA), measured after an 8–12 h fast to exclude dietary bias, independently correlates with cardiometabolic disorders and frailty, serving as a reliable geriatric risk biomarker (4–6).

In this cross-sectional study, nursing home residents were a particularly important group for investigating the FSUA–AD relationship for various reasons. First, the prevalence of AD and its associated comorbidities was significantly higher in long-term care settings than in older adults living in the community (7, 8). These conditions not only elevate the baseline risk of cognitive decline but also directly affect uric acid metabolism by altering renal excretion, oxidative stress, and nutritional status. Second, institutionalized care provides a controlled environment with standardized dietary and medication management, thereby reducing the confounding variables commonly encountered in community-based research. Third, despite their high vulnerability, nursing home residents are underrepresented in AD biomarker research. The evidence obtained from this group may provide complementary insights into the relationship between uric acid levels and cognitive function within the context of multimorbidity and cognitive decline (9, 10).

Uric acid (UA), the final metabolite of purine metabolism, has piqued interest because of its potential involvement in neurodegenerative diseases (11, 12). Traditionally perceived as a potent antioxidant, elevated UA levels in serum have been hypothesized to counteract oxidative stress, a mechanism implicated in AD pathophysiology (13–15). However, an increasing body of evidence indicates that UA may also exhibit pro-inflammatory and pro-oxidant characteristics under certain conditions, potentially contributing to endothelial dysfunction, neuroinflammation, and amyloidogenesis (16–18). UA intersects with multiple pathogenic hallmarks of neurodegeneration, including mitochondrial dysfunction, lipid peroxidation, and ferroptosis, which have been increasingly implicated in the metabolic-inflammatory cascade underlying AD. This broader framework suggests that UA may modulate neurodegenerative risk not only through oxidative stress, but also through its interactions with neuroinflammatory and metabolic dysregulation (19). The paradoxical characteristics of UA have resulted in conflicting findings. Some studies, particularly those examining specific subgroups such as elderly patients, indicated that elevated UA levels may have potentially harmful effects (20–22). Moreover, research focusing on vulnerable nursing home populations is limited, with existing studies frequently lacking rigorous control of numerous confounding variables present in this setting. Considering the contradictory biological effects of UA and the paucity of context-specific data on nursing home residents, we investigated the association between FSUA levels and AD. These findings offer a more nuanced understanding of this complex relationship and may inform future research.

## 2 Materials and methods

### 2.1 Data source and study population

**Figure 1:**
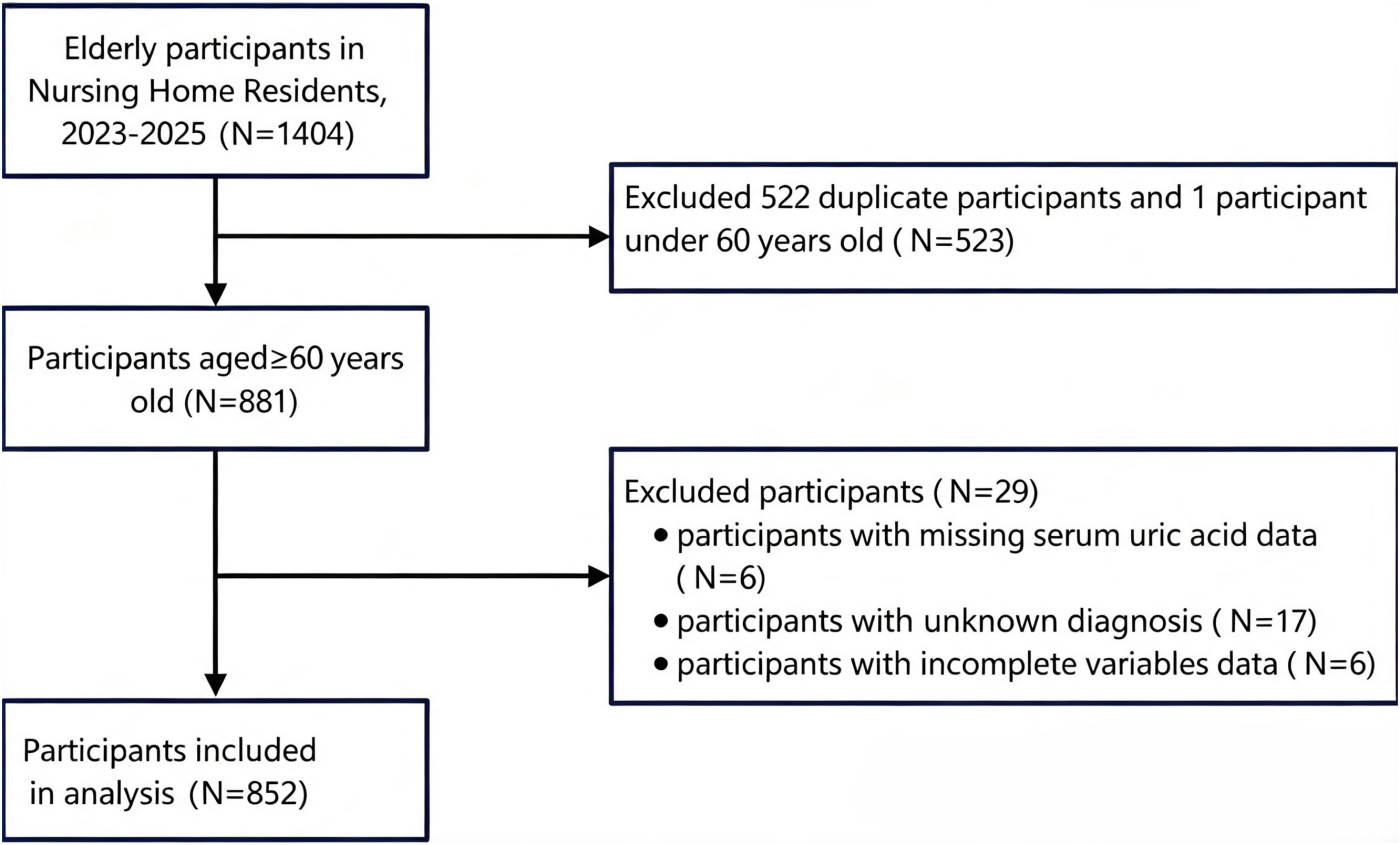
Flowchart of the screening and enrollment of study participants.

This retrospective cross-sectional study included elderly individuals who underwent physical examinations at a nursing home in Guangzhou between August 2023 and June 2025. Guangzhou Geriatric Hospital and Guangzhou Nursing Home operate within the same facility and provide a comprehensive range of services to meet community needs, including medical care, rehabilitation training, psychological counseling, social work services, assistance with daily living, and cultural entertainment. Participants were excluded if they were younger than 60 years, had duplicate data entries, lacked serum UA data, had unclear diagnoses, or had incomplete data. Figure 1 shows the exclusion criteria. The final analysis included data from 852 participants. This study complied with the Declaration of Helsinki and was approved by the Ethics Review Committee of Guangzhou Geriatric Hospital. The requirement for informed consent was waived because this study used anonymized retrospective data.

### 2.2 FSUA

Serum UA levels were determined using an automatic biochemical analyzer (7180 Clinical Analyzer, Hitachi, Ltd., Tokyo, Japan) and a UA detection reagent (Medicalsystem Biotechnology Co., LTD., Ningbo, Zhejiang, China). After fasting overnight, trained staff at Guangzhou Geriatric Hospital collected and analyzed the participants' blood samples following standard procedures using an enzymatic colorimetric test to measure serum UA levels.

### 2.3 AD

Determination of AD status was based on clinical diagnoses documented in the medical records of participating nursing homes conducted during routine clinical visits by attending neurologists or geriatricians. The diagnostic evaluation process typically encompasses comprehensive history taking, physical and neurological examinations, assessment of cognitive and functional decline (e.g., activities of daily living) via information provided by an informant, and utilization of imaging and laboratory testing to corroborate the diagnosis. In this study, the clinical diagnosis of AD was established by clinicians in conjunction with real-world clinical scenarios, adhering to the diagnostic criteria set forth by the National Institute on Aging and Alzheimer's Disease Association (NIA-AA) in 2018. Cases of AD and dementia were identified by searching for codes from the 10th revision of the International Classification of Diseases (ICD-10) within the electronic medical records. Medical records were meticulously reviewed for each identified case and the diagnosis was confirmed by a specialist. Other potential causes of cognitive impairment have been thoroughly investigated within the constraints of current clinical conditions. These conditions include cerebrovascular disease, Parkinson's disease, frontotemporal dementia, dementia with Lewy bodies, normal-pressure hydrocephalus, and thyroid dysfunction. Participants who did not have a recorded diagnosis of dementia or were diagnosed with other dementia subtypes (such as vascular dementia, frontotemporal dementia, and dementia with Lewy bodies) without concurrent coding for Alzheimer's disease were consistently categorized into the non-AD group (non-AD). Participants with incomplete diagnostic records or ambiguous diagnostic conclusions were excluded from the primary analysis.

### 2.4 Covariates

Confounding variables were selected based on clinical expertise, existing research, the significance of covariates in univariate analyses, and any correlations with the outcomes of interest or shifts in the effect estimate greater than 10% (23). A broad spectrum of potential covariates were evaluated, including sex, age, body mass index (BMI), education level, cerebrovascular disease, coronary heart disease, diabetes mellitus, hypertension, number of medications, systolic blood pressure (SBP), regular exercise, fasting blood glucose (FBG), hemoglobin (HB), platelet count (PLT), total bilirubin (TBIL), low-density lipoprotein cholesterol (LDL-C), and high-density lipoprotein cholesterol (HDL-C). Participants were categorized into two age groups: less and more than 80 years. BMI was determined by dividing weight by height squared (kg/m^2^). The “Dietary Guidelines for the Elderly in China (2022)” core recommendations classify elderly BMI into three categories, with a normal BMI standard set between 20 and 26.9 kg/m^2^ (24). Education level was categorized into two groups: less than or equal to sixth grade and above sixth grade. Two groups were formed based on HB levels: one with less than 120 g/L and the other with 120 g/L or more. Using the CKD-EPI formula, the estimated glomerular filtration rate (eGFR) was estimated for each participant by factoring in age, sex, and serum creatinine levels (25). The participants were divided into four groups based on their use of urate-lowering medications and diuretics: category 0 (neither urate-lowering drugs nor diuretics used), category 1 (only urate-lowering drugs used), category 2 (only diuretics used), and category 3 (both urate-lowering drugs and diuretics used).

### 2.5 Analysis of statistics

Continuous variables were analyzed using the independent sample *t*-test and presented as means ± standard deviation (SD). The chi-square test was used to compare categorical variables, which are shown as counts and percentages [*n* (%)]. The analysis of skewed continuous variables was conducted using the Mann–Whitney *U*-test, which is summarized as the median with interquartile range (IQR). To explore the association between FSUA levels and the likelihood of AD, multivariate logistic regression analysis was used to estimate the odds ratios (OR) and 95% confidence intervals (CI). Unadjusted and multivariable-adjusted models were used. Four models were constructed for analysis, and Model 1 was implemented without adjusting for covariates. Model 2: Factoring for sex, age, BMI, and educational level. Adjustments for cerebrovascular disease, coronary heart disease, diabetes, hypertension, and the number of medications were added to Model 3, which was built on Model 2. Finally, Model 4 (the main model) incorporated SBP, regular exercise, FBG, HB, PLT, TBIL, eGFR, type of medication, and LDL-C and HDL-C, based on Model 3. Subgroup analyses were performed based on predefined variables, and the interactions among these subgroups were assessed using the likelihood ratio test. To investigate the dose-response association between FSUA and AD, we applied a three-node restricted cubic spline (RCS) regression model and accounted for the variables present in the logistic regression model 4. Statistical analyses were performed using R software (version 4.2.2, https://www.R-project.org/, The R Foundation, Vienna, Austria) and Free Statistics software version 2.3. Statistical analyses were two-sided, and *p* < 0.05 was considered statistically significant.

## 3 Results

### 3.1 Characteristics and outcomes

Elderly individuals residing in nursing homes who volunteered for physical examination between August 2023 and June 2025 were included in this study. The screening process commenced with the exclusion of 522 duplicate entries and one individual under the age of 60 years, resulting in 881 participants who satisfied the age criterion of 60 years or older. Subsequently, 29 participants were excluded because of incomplete data on FSUA levels, ambiguous diagnoses of AD, or incomplete information on key variables. Consequently, the final analytical sample comprised 852 participants, 196 of whom had AD, as shown in Figure 1. In this cross-sectional study, 852 nursing home residents were stratified into quartiles based on their FSUA levels. Table 1 provides a detailed overview of participants' baseline characteristics. The mean age of those participating was 81.0 ± 8.8 years, and no differences were observed across FSUA quartiles (*p* = 0.6). The study population was predominantly comprised females (66.3%), and the distribution of sex demonstrated variation across groups (*p* = 0.001), with an increasing proportion of males observed from the lowest to the highest FSUA quartile. BMI exhibited an upward trend from the first to fourth quartile (*p* < 0.001). Several metabolic parameters, such as total bilirubin (*p* < 0.001), LDL-C, and HDL-C, and prevalence of hypertension and diabetes mellitus (*p* < 0.05), and displayed differences across quartiles (Supplementary Table S1). Conversely, factors including regular exercise habits, HB, cerebrovascular disease, and coronary heart disease did not vary among the groups (*p* > 0.05).

**Table 1:** Baseline characteristics of participants.

Variables	Fasting serum uric acid levels (FSUA, μmol/L)					*p-*value
	Total (*n* = 852)	Q1 (*n* = 211)	Q2 (*n* = 213)	Q3 (*n* = 215)	Q4 (*n* = 213)	
Sex, *n* (%)						0.001
Male	287 (33.7)	58 (27.5)	59 (27.7)	79 (36.7)	91 (42.7)	
Female	565 (66.3)	153 (72.5)	154 (72.3)	136 (63.3)	122 (57.3)	
Age (year)	81.0 ± 8.8	80.7 ± 9.3	80.7 ± 8.4	81.7 ± 8.2	81.1 ± 9.2	0.6
BMI (kg/m^2^)	22.0 ± 4.5	20.1 ± 4.7	21.7 ± 4.3	22.6 ± 4.2	23.6 ± 4.1	< 0.001
Education level (year), *n* (%)						0.653
≤ 6	565 (66.3)	141 (66.8)	137 (64.3)	139 (64.7)	148 (69.5)	
>6	287 (33.7)	70 (33.2)	76 (35.7)	76 (35.3)	65 (30.5)	
Regular exercise, *n* (%)						0.8
No/occasional	540 (63.4)	135 (64)	129 (60.6)	138 (64.2)	138 (64.8)	
Yes	312 (36.6)	76 (36)	84 (39.4)	77 (35.8)	75 (35.2)	
Self-care ability, *n* (%)						< 0.001
Independent	328 (39.8)	46 (22.4)	86 (41.7)	101 (48.1)	95 (46.6)	
Partially dependent	209 (25.3)	49 (23.9)	52 (25.2)	46 (21.9)	62 (30.4)	
Completely dependent	288 (34.9)	110 (53.7)	68 (33)	63 (30)	47 (23)	
HB (g/L)	122.6 ± 18.3	120.3 ± 19.5	123.0 ± 16.3	124.4 ± 17.2	122.8 ± 19.8	0.142
SBP (mmHg)	127.0 ± 16.8	122.2 ± 15.6	126.9 ± 17.0	129.2 ± 16.9	129.7 ± 16.9	< 0.001
DBP (mmHg)	73.3 ± 9.5	72.8 ± 9.4	73.8 ± 9.9	72.8 ± 9.3	73.8 ± 9.3	0.526
Hypertension, *n* (%)						< 0.001
No	250 (29.3)	79 (37.4)	73 (34.3)	53 (24.7)	45 (21.1)	
Yes	602 (70.7)	132 (62.6)	140 (65.7)	162 (75.3)	168 (78.9)	
Diabetes mellitus, *n* (%)						< 0.001
No	615 (72.2)	172 (81.5)	155 (72.8)	153 (71.2)	135 (63.4)	
Yes	237 (27.8)	39 (18.5)	58 (27.2)	62 (28.8)	78 (36.6)	
Cerebrovascular disease, *n* (%)						0.892
No	521 (61.2)	128 (60.7)	135 (63.4)	129 (60)	129 (60.6)	
Yes	331 (38.8)	83 (39.3)	78 (36.6)	86 (40)	84 (39.4)	
Coronary heart disease, *n* (%)						0.307
No	615 (72.2)	163 (77.3)	151 (70.9)	151 (70.2)	150 (70.4)	
Yes	237 (27.8)	48 (22.7)	62 (29.1)	64 (29.8)	63 (29.6)	
Number of medications, *n* (%)						0.015
0	101 (11.9)	34 (16.1)	31 (14.6)	16 (7.4)	20 (9.4)	
1–4	439 (51.5)	108 (51.2)	93 (43.7)	119 (55.3)	119 (55.9)	
≥5	312 (36.6)	69 (32.7)	89 (41.8)	80 (37.2)	74 (34.7)	
Type of medications, *n* (%)						0.012
0	749 (87.9)	190 (90)	195 (91.5)	192 (89.3)	172 (80.8)	
1	23 (2.7)	7 (3.3)	4 (1.9)	6 (2.8)	6 (2.8)	
2	75 (8.8)	14 (6.6)	12 (5.6)	15 (7)	34 (16)	
3	5 (0.6)	0 (0)	2 (0.9)	2 (0.9)	1 (0.5)	
MMSE score	20.0 (9.0, 25.0)	11.5 (0.0, 23.0)	20.0 (11.0, 25.0)	21.0 (10.8, 25.0)	22.0 (14.0, 25.0)	< 0.001
eGFR	85.7 (65.9, 109.3)	112.3 (90.2, 146.4)	89.2 (75.8, 110.3)	80.1 (62.6, 97.6)	68.3 (52.6, 83.9)	< 0.001

The data are shown as the mean ± standard deviation, median (interquartile range), or frequency (%), as appropriate.

BMI, body mass index; SBP, systolic blood pressure; DBP, diastolic blood pressure; HB, hemoglobin; Number of medications, number of concomitant medications; MMSE, mini-mental state examination; eGFR, estimated glomerular filtration rate; Regular exercise was defined as engaging in exercise more than once per week for a minimum duration of 1 year; Self-care ability was assessed using the Chinese Ability of Daily Life Self-care Scale. Based on the established cutoff values, participants were categorized into three ordinal groups: Independent (score 0–3), Partially dependent (score 4–18, combining the original mild and moderate dependency categories), and completely dependent (score ≥ 19); Type of medications: participants were divided into four groups based on their use of urate-lowering medications and diuretics: Category 0 (neither urate-lowering drugs nor diuretics used), Category 1 (only urate-lowering drugs used), Category 2 (only diuretics used), and Category 3 (both urate-lowering drugs and diuretics used).

Q1–Q4, Quartile according to Fasting Serum Uric Acid Levels.

### 3.2 Association between FSUA and AD

**Figure 2:**
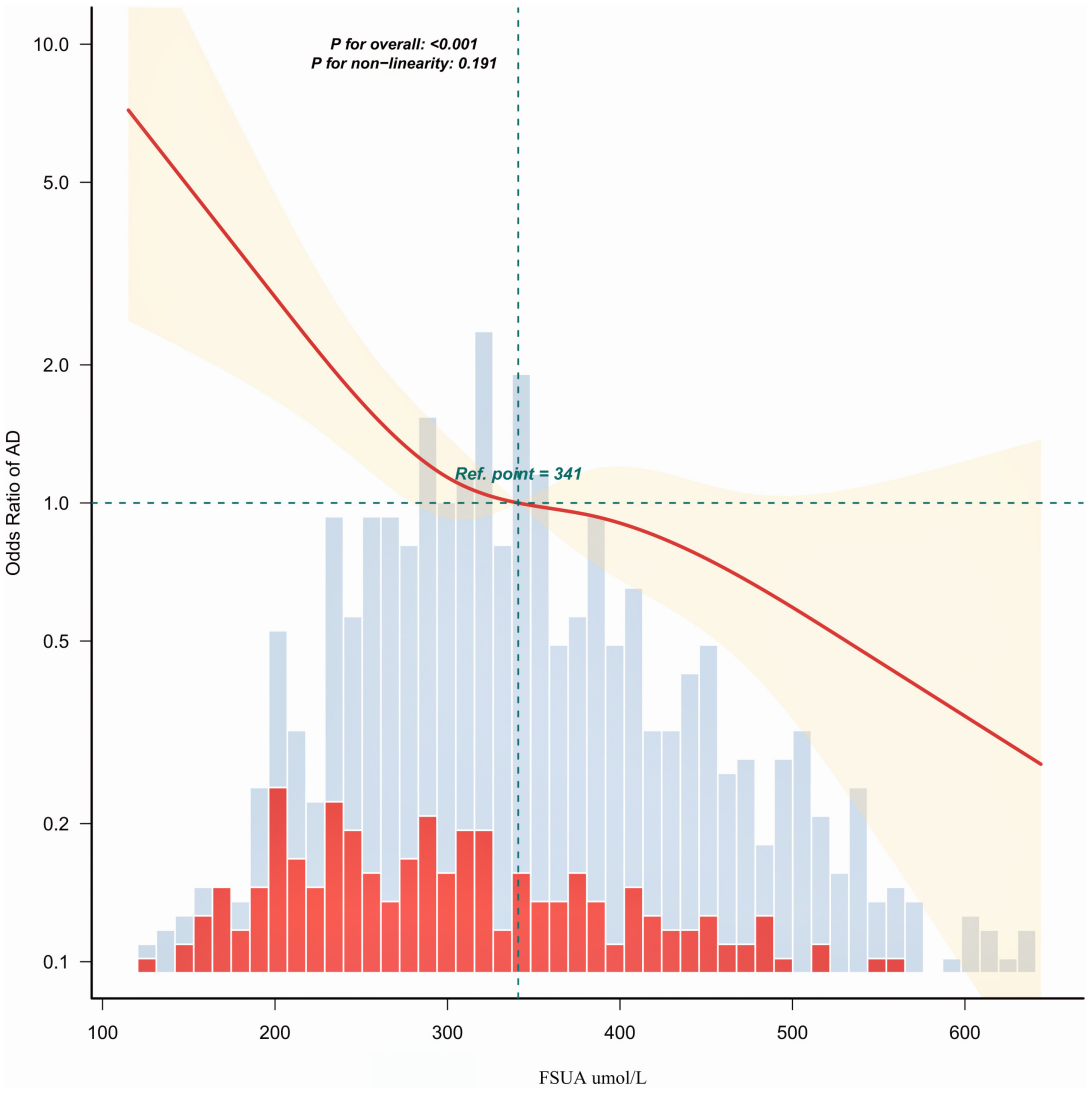
Odds ratios for the association between FSUA and AD. The lines represent adjusted odds ratios (solid line) and 95% confidence intervals (long dashed lines). Reference value 341 is the median FSUA. Histograms represent the distribution of FSUA in participant with and without AD, with blue indicating no AD and red indicating AD. Adjusted for sex, age, BMI, and education level cerebrovascular disease, coronary heart disease, diabetes mellitus, hypertension, regular exercise, SBP, FBG, HB, PLT, TBIL, eGFR, LDL-C, HDL-C, number of medications, type of medications. FSUA, Fasting Serum Uric Acid; AD, Alzheimer's disease; BMI, body mass index; SBP, systolic blood pressure; HB, hemoglobin; PLT, platelet; TBIL, total bilirubin; LDL-C, low-density lipoprotein cholesterol; HDL-C, high-density lipoprotein cholesterol; Number of medications, number of concomitant medications; eGFR, estimated glomerular filtration rate; FBG, fasting blood glucose. Regular exercise was defined as engaging in exercise more than once per week for a minimum duration of 1 year. Only 99% of the data are shown; Type of medications: participants were divided into four groups based on their use of urate-lowering medications and diuretics: Category 0 (neither urate-lowering drugs nor diuretics used), Category 1 (only urate-lowering drugs used), Category 2 (only diuretics used), and Category 3 (both urate-lowering drugs and diuretics used).

Researchers employed multivariable logistic regression to explore the association between FSUA levels and the odds of developing AD. When FSUA was treated as a continuous variable, a 10 μmol/L increment in FSUA was linked to 5% lower odds of AD (95% CI, 0.93–0.98; *p* < 0.001). Similarly, when FSUA levels were divided into quartiles, the higher quartiles demonstrated progressively lower odds of developing AD than the lowest quartile (Q1). In Model 4, the odds ratios were 0.54 (95% CI, 0.33–0.89; *p* = 0.015) for the second quartile, 0.42 (95% CI, 0.25–0.71; *p* = 0.001) for the third quartile (Q3), and 0.36 (95% CI, 0.19–0.66; *p* = 0.001) for the fourth quartile (Q4), with a trend observed (*p* for trend < 0.001). After accounting for potential confounders, such as sex, age, BMI, education, comorbidities, and laboratory parameters, the findings consistently indicated a negative correlation between FSUA levels and the likelihood of developing AD. Table 2 lists the detailed results. Analysis using a restricted triple spline revealed a linear decrease in the odds of developing AD with elevated FSUA levels (Figure 2).

**Table 2:** Association between FSUA and AD.

Variable total no (%)			Model 1		Model 2		Model 3		Model 4	
			OR (95%CI)	*p-*value	OR (95%CI)	*p-*value	OR (95%CI)	*p-*value	OR (95%CI)	*p-*value
FSUA (10 μmol/L)	852	196 (23)	0.94 (0.92~0.96)	< 0.001	0.95 (0.93~0.97)	< 0.001	0.95 (0.93~0.97)	< 0.001	0.95 (0.93~0.97)	< 0.001
FSUA (quartile)										
Q1 (115, 274)	211	82 (38.9)	1 (Ref)		1 (Ref)		1 (Ref)		1 (Ref)	
Q2 (275, 341)	213	49 (23)	0.47 (0.31~0.72)	< 0.001	0.51 (0.33~0.8)	0.003	0.54 (0.34~0.86)	0.009	0.54 (0.33~0.89)	0.015
Q3 (342, 421)	215	39 (18.1)	0.35 (0.22~0.54)	< 0.001	0.41 (0.26~0.66)	< 0.001	0.43 (0.26~0.7)	0.001	0.42 (0.25~0.71)	0.001
Q4 (422, 846)	213	26 (12.2)	0.22 (0.13~0.36)	< 0.001	0.29 (0.17~0.5)	< 0.001	0.35 (0.2~0.61)	< 0.001	0.36 (0.19~0.66)	0.001
*p* for trend				< 0.001		< 0.001		< 0.001		< 0.001

Model 1: Non-adjusted.

Model 2: Adjusted for sex, age, BMI, and education level.

Model 3: Adjusted for Model 2+ cerebrovascular disease, coronary heart disease, diabetes mellitus, hypertension, and number of medications.

Model 4: Adjusted for Model 3+SBP, regular exercise, FBG, HB, PLT, TBIL, LDL-C, HDL-C, eGFR, and type of medications.

FSUA, Fasting Serum Uric Acid; AD, Alzheimer's disease; BMI, body mass index; SBP, systolic blood pressure; HB, hemoglobin; WBC, white blood cell; PLT, platelet; TBIL, total bilirubin; LDL-C, low-density lipoprotein cholesterol; HDL-C, high-density lipoprotein cholesterol; Number of medications, number of concomitant medications; TC, total cholesterol; MMSE, mini-mental state examination; eGFR, estimated glomerular filtration rate; FBG, fasting blood glucose; ALT, alanine aminotransferase; AST, aspartate aminotransferase; Regular exercise was defined as engaging in exercise more than once per week for a minimum duration of 1 year; Self-care ability was assessed using the Chinese Ability of Daily Life Self-care Scale. Based on the established cutoff values, participants were categorized into three ordinal groups: Independent (score 0–3), Partially dependent (score 4–18, combining the original mild and moderate dependency categories), and completely dependent (score ≥19); Type of medications, participants were divided into four groups based on their use of urate-lowering medications and diuretics: Category 0 (neither urate-lowering drugs nor diuretics used), Category 1 (only urate-lowering drugs used), Category 2 (only diuretics used), and Category 3 (both urate-lowering drugs and diuretics used).

Q1–Q4, Quartile according to Fasting Serum Uric Acid Levels. No, sample size; OR, odds ratio; Cl, confidence interval; Ref, reference.

### 3.3 Subgroup analyses

**Figure 3:**
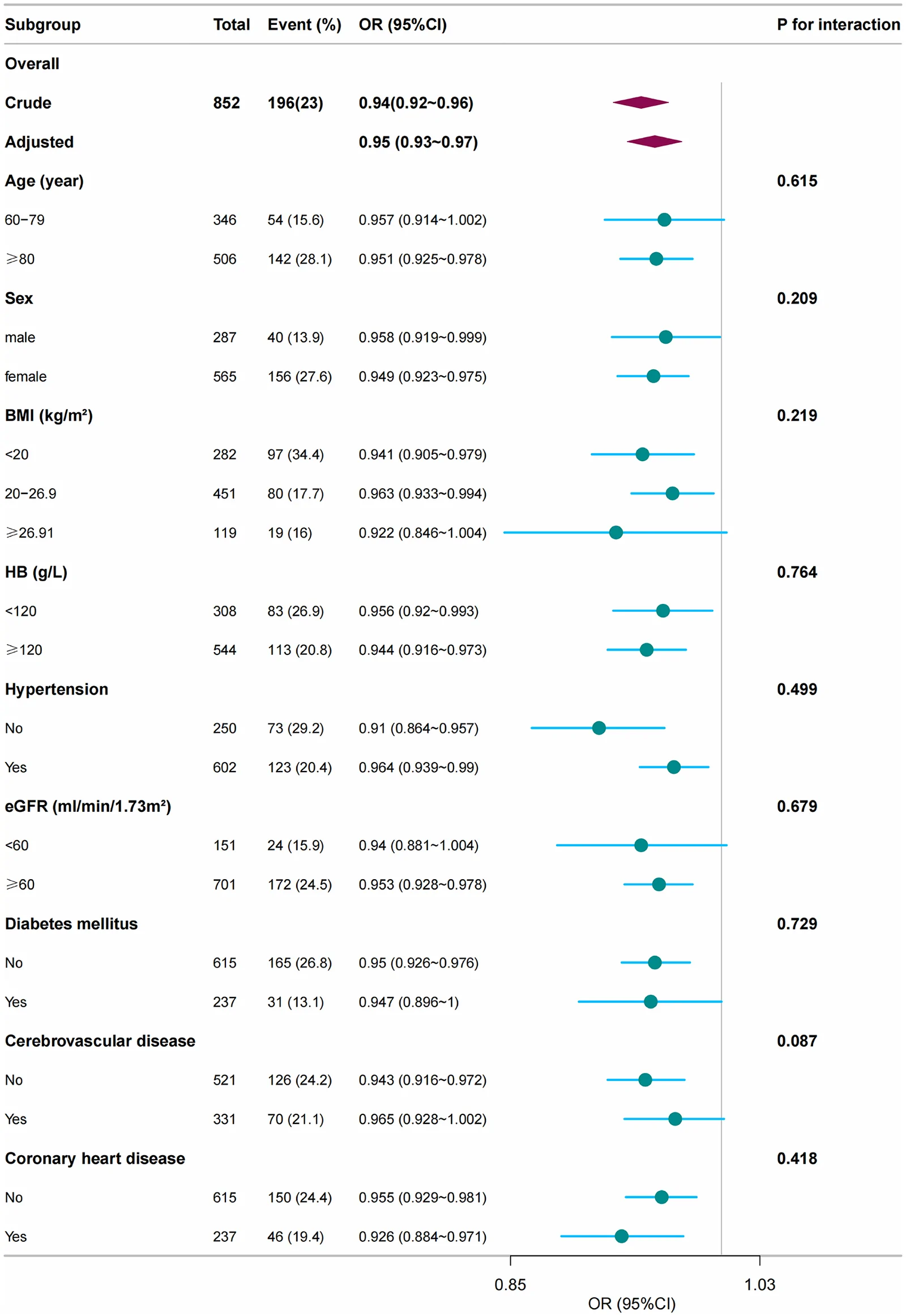
Forest plot of multivariable logistic analysis between FSUA and AD. FSUA, Fasting Serum Uric Acid; AD, Alzheimer's disease; HB, hemoglobin; CI, confidence interval; OR, odds ratio; BMI, body mass index. The vertical solid line represents OR = 1.

Figure 3 demonstrates stability in all subgroups when the data were stratified by age, sex, BMI, hypertension, diabetes mellitus, cerebrovascular disease, and eGFR.

## 4 Discussion

To the best of our knowledge, we consistently observed an inverse relationship between FSUA levels and AD in this retrospective, cross-sectional study. The findings demonstrated consistent results across diverse subgroup analyses, with stable associations evident in both the unadjusted models and models with stepwise covariate adjustment.

AD, the most frequently occurring type of dementia, is a significant and growing global public health concern, particularly among the rapidly aging population in nursing homes (26). Our primary finding of an inverse association between SUA levels and the occurrence of AD is consistent with that of previous studies. A large-scale prospective cohort study demonstrated that individuals in the highest SUA quintile of SUA had a 25% reduced risk of developing dementia compared to those in the lowest quintile (27). The Regal 2.0 project study also discovered that SUA levels were notably reduced in patients with AD compared to healthy individuals, indicating that UA levels were inversely associated with AD in older adults (15). This finding aligns with the outcomes of another systematic review and meta-analysis that suggested that low levels of UA might pose a risk for AD and dementia related to Parkinson's disease (PD) (28). Furthermore, research on UA and AD-related cerebral changes has indicated a significant correlation between low UA levels and AD-related brain hypometabolism, although the causality of this relationship requires further investigation (29). In addition to cognitive outcomes, recent evidence indicates that systemic biomarkers may be associated with structural brain alterations integral to Alzheimer's pathology. For example, increased erythrocyte levels in the cerebrospinal fluid have been associated with hippocampal atrophy, a key neuroimaging characteristic of AD, underscoring the potential link between peripheral biological markers and clinically significant neurodegeneration (30). These findings further underscore the importance of examining peripheral biomarkers such as serum UA in relation to AD-related structural brain changes. Another study supports this by finding a positive association between cognitive function and UA levels, particularly in the later stages of AD, suggesting a potential link between UA metabolism and the AD pathological cascade by influencing β-amyloid (Aβ42) and its downstream pathological cascade (31). The key strength of this study lies in its focus on a large high-risk nursing home population. Four sequentially adjusted multivariate models were used. Analyses using continuous, categorical, and RCS models consistently revealed an inverse association between FSUA and AD, supporting the reliability and credibility of our findings.

However, despite observational studies proposing the neuroprotective effects of UA, Mendelian randomization studies have not verified a causal link between UA and AD. These studies reported that although a *U*-shaped association was observed between brain disease and uric acid, the relationship was not causal (32). Furthermore, another bidirectional Mendelian randomization study did not establish a causal association between UA levels and the risk of AD and found a link between higher UA levels and a greater risk of AD (33). Several factors may have accounted for these discrepancies. One potential explanation for this is the antioxidant properties of UA, which may contribute to its protective effects against AD. Research indicates that UA can decelerate cognitive decline to some extent through its antioxidant capacity, particularly in subgroups with slight cognitive impairment and dementia (34). Moreover, UA may also plays a protective role in cognitive decline by mitigating the deleterious effects of biomarkers associated with AD, such as Aβ1–42 and tau protein (34). UA confers neuroprotection through its antioxidant properties; however, it may simultaneously exert deleterious effects on the cardiovascular system owing to its pro-oxidative characteristics (11). This duality may explain the inconsistent findings reported in the literature. An increase in UA levels has been associated with an increased risk of cardiovascular disease, which is a recognized risk factor for AD (35). Thus, UA may play a complex role in AD, with both protective and harmful effects. Compared with previous studies, the present study was a retrospective cross-sectional study based on 852 elderly individuals in a single nursing home, focusing on the cross-sectional association between serum UA and prevalent AD rather than the incidence, cognitive decline or biological mechanisms. The study population, design, and outcome measures were distinctly different from those of Ye et al. (34), Alam et al. (35), and Tovchiga and Inkielewicz-Stepniak (11), providing supplementary evidence for the UA–AD relationship in the special elderly population of nursing homes.

Consequently, the involvement of UA in AD is likely to be multifaceted, encompassing both protective and potentially harmful effects. These findings are consistent with those of previous studies. We consistently observed that higher FSUA levels were inversely related to the occurrence of AD, even after adjusting for confounding variables, such as age, BMI, educational level, sex, and baseline health conditions. Further research is required to validate these findings and elucidate the complex relationships and underlying mechanisms. Interestingly, there was a significant association between FSUA levels and AD, and this association remained consistent across subtypes, including sex, age, BMI, baseline disease, education level, and number of medications.

Our study had several strengths. The information was obtained directly from a hospital specializing in geriatric care. This methodological approach ensured the inclusion of a population at high risk for cognitive disorders associated with AD, thereby enhancing the clinical relevance of our findings. Consequently, the results of this study have significantly practical value for clinicians, particularly geriatricians and neurologists. Furthermore, given the inconsistencies in conclusions across existing studies, this study offers fresh clinical insights into the crucial scientific question regarding the involvement of SUA in AD. It also provides a theoretical foundation for investigating serum UA or its precursors as potential biomarkers or targets for intervention.

This study has several limitations owing to its cross-sectional design, which hinders the establishment of a definitive causal temporal relationship between FSUA and AD. It remains unclear whether reduced FSUA serves as a preclinical risk factor preceding the onset of AD or whether it merely represents a secondary biological consequence of established AD, making reverse causation a plausible alternative explanation. Furthermore, although the clinical diagnosis of AD was determined through thorough evaluation and confirmation by specialists in accordance with the NIA-AA 2018 criteria, it was primarily based on routine clinical diagnoses rather than on standardized research-grade protocols. This approach to clinical diagnosis may have resulted in a residual misclassification of AD and other dementia subtypes that share overlapping clinical features. If such misclassification occurred, it would likely bias the observed associations between FSUA and AD toward the null owing to the heterogeneity in UA metabolism across various dementia etiologies. Various pathophysiological disruptions inherent to the progression of AD, such as metabolic dysfunction, progressive weight loss, malnutrition, and systemic low-grade inflammation, may independently contribute to the reduction in serum UA levels and confound the observed cross-sectional correlation (36). Consequently, future prospective cohort studies and Mendelian randomization analyses are necessary to rigorously validate the causal relationship and clarify the temporal sequence underlying this association.

In addition, despite adjusting for a range of known confounders, such as age, sex, BMI, and underlying disease, there is still the possibility of residual confounding by unmeasured or unknown variables, such as albumin, prealbumin, nutritional risk score, and frailty index, which may affect UA levels and odds of AD. Future prospective studies are recommended to include comprehensive nutritional assessment indicators, serum prealbumin or handgrip strength to verify the conclusions of this study. Furthermore, the data were obtained from a single geriatric hospital, which potentially limits the representativeness of the study sample. Future studies should include multicenter community-based validations among various populations to improve the generalizability of our results.

## 5 Conclusion

In this cross-sectional study involving a nursing home population, FSUA levels were negatively associated with the prevalence of AD. Prospective and mechanistic studies are warranted to clarify causality and underlying biological interpretations.

## Data Availability

The data analyzed in this study is subject to the following licenses/restrictions: the dataset supporting this study contains anonymized clinical and laboratory data from nursing home residents. Due to patient privacy regulations and institutional data governance policies, the dataset is not publicly available. Access to the de-identified data may be granted to qualified researchers upon reasonable request to the corresponding author, subject to institutional review board approval and completion of a data use agreement to ensure compliance with ethical and privacy standards. Requests to access these datasets should be directed to WZ, zwjzwj223223@163.com.
