# Supplementary material for: Association between fasting serum uric acid levels and Alzheimer's disease in nursing home residents: a cross-sectional study
Source: Front Nutr. 2026 Aug 6;13:1878852. doi: 10.3389/fnut.2026.1878852 (PMC13492062; doi:10.3389/fnut.2026.1878852)
Supplement: Supplementary file 1 [file Table_1.docx]

Supplementary Material

# 1 Supplementary Figures and Tables

## 1.1 Supplementary Tables

**Supplementary Table 1.** The figure legends are required to have the same font as the main text, 12-point normal Times New Roman, single spaced. Please use a single paragraph for each legend and prepare the figures keeping in mind the PDF layout.

Supplementary table S1. Baseline characteristics of participants.

| **Variables** | **Fasting Serum Uric Acid Levels (FSUA, μmol/L)** | | | | | ***p* value** |
| --- | --- | --- | --- | --- | --- | --- |
|  | **Total**  **(n = 852)** | **Q1**  **(n = 211)** | **Q2**  **(n = 213)** | **Q3**  **(n = 215)** | **Q4**  **(n = 213)** |  |
| WBC (10^9^/L) | 6.7 ± 1.8 | 6.5 ± 1.9 | 6.4 ± 1.7 | 6.8 ± 1.8 | 7.2 ± 1.8 | < 0.001 |
| TBIL (μmol/L) | 10.2 ± 4.8 | 9.5 ± 4.3 | 10.1 ± 4.5 | 11.4 ± 6.0 | 9.7 ± 4.1 | < 0.001 |
| LDL-C (mmol/L) | 2.9 ± 1.0 | 2.7 ± 1.0 | 2.8 ± 1.0 | 2.8 ± 0.9 | 3.1 ± 0.9 | 0.003 |
| HDL-C (mmol/L) | 1.5 ± 0.4 | 1.5 ± 0.4 | 1.5 ± 0.4 | 1.5 ± 0.3 | 1.4 ± 0.3 | < 0.001 |
| Scr (μmol/ L) | 82.6 ± 47.6 | 61.0 ± 21.3 | 76.3 ± 48.0 | 85.9 ± 39.5 | 106.9 ± 60.6 | < 0.001 |
| FBG (mmol/ L) | 5.7 ± 2.2 | 5.5 ± 2.8 | 5.7 ± 2.1 | 5.7 ± 1.9 | 6.0 ± 2.0 | 0.188 |
| PLT (10^9^/ L) | 234.3 ± 75.5 | 256.2 ± 93.2 | 226.4 ± 59.5 | 224.0 ± 71.5 | 230.9 ± 70.1 | < 0.001 |
| TC (mmol/L) | 4.6 (3.8, 5.3) | 4.3 (3.7, 5.2) | 4.6 (3.9, 5.4) | 4.6 (3.8, 5.3) | 4.6 (4.0, 5.4) | 0.185 |
| ALT (U/L) | 16.0 (11.0, 22.0) | 15.0 (11.0, 22.0) | 15.0 (11.0, 21.0) | 17.0 (12.0, 23.0) | 16.0 (11.0, 23.0) | 0.546 |
| AST (U/L) | 22.0 (18.0, 26.0) | 22.0 (17.5, 26.0) | 22.0 (19.0, 25.0) | 23.0 (19.0, 27.0) | 22.0 (18.0, 27.0) | 0.259 |

The data are shown as the mean ± standard deviation, median (interquartile range), or frequency (%), as appropriate.

Abbreviations: WBC: white blood cell; PLT: platelet; TBIL: total bilirubin; LDL-C: low-density lipoprotein cholesterol; HDL-C: high-density lipoprotein cholesterol; TC: total cholesterol; MMSE: mini-mental state examination; Scr: serum creatinine; FBG: fasting blood glucose; ALT: alanine aminotransferase; AST: aspartate aminotransferase;

Q1-Q4: Quartile according to Fasting Serum Uric Acid Levels.
